# Central Nervous System Manifestations of Long COVID: A Systematic Review

**DOI:** 10.7759/cureus.83247

**Published:** 2025-04-30

**Authors:** Naga Vijaya Lakshmi Divya Boorle, Nithin C Kurra, Nikhila Gandrakota, Karnav Modi, Kavya Sudireddy, Shayan A Irfan, Akhil Jain, Priyanka A Parikh, Dinesh Jillella

**Affiliations:** 1 Department of Family Medicine and Public Health Sciences, Wayne State University School of Medicine, Detroit, USA; 2 Department of Neurology, University of Nebraska Medical Center, Omaha, USA; 3 Department of Family and Preventative Medicine, Emory University School of Medicine, Atlanta, USA; 4 Department of Internal Medicine, University of Missouri - Kansas City School of Medicine, Kansas City, USA; 5 Department of Internal Medicine, UMass (University of Massachusetts) Chan Medical School, Worcester, USA; 6 Department of Internal Medicine, Dow University of Health Sciences, Karachi, PAK; 7 Department of Internal Medicine, Mercy Catholic Medical Center, Darby, USA; 8 Department of Pediatrics, Pramukhswami Medical College, Karamsad, IND; 9 Department of Neurology, Emory University School of Medicine, Atlanta, USA

**Keywords:** long covid, long-term, memory impairment, neurological manifestations, neuropsychiatric manifestations, olfactory dysfunction, post-covid-19 fatigue

## Abstract

Severe acute respiratory syndrome coronavirus-2 (SARS-CoV-2) has been one of the most widespread and devastating global pandemics, impacting hundreds of millions of people worldwide. After the cessation of active infection, the disease continues to have a disabling impact due to the persistence of fatigue, brain fog, anxiety, and depression - among the most common symptoms. This study explores the progression of neurological symptoms over 12 months and beyond following an initial diagnosis of COVID-19. Through an electronic search of eligible studies from PubMed, the Cochrane Trial Register, and Google Scholar, 10 studies were included for qualitative analysis. The systematic review highlights the similarities and differences in findings across the included studies. Olfactory dysfunction was prevalent in 0.9%-51% of individuals, and taste impairment was observed in 1.1%-21.3%. At 12 months, anxiety was more prevalent (3.5%-29%) than depression (3.5%-26%). Fatigue was the predominant neurocognitive complaint in 56% of individuals with severe COVID-19. Nearly half of individuals reported sleep difficulties. Memory impairment, followed by headaches and dizziness, also constitutes neurocognitive symptoms reported at 12 months. Our study found that there is a significant neurological burden one year following the diagnosis of COVID-19. Further studies exploring the pathological mechanisms of long-term COVID-19 are necessary to better delineate the mechanisms behind several long-term neurological manifestations of COVID-19.

## Introduction and background

Severe acute respiratory syndrome coronavirus-2 (SARS-CoV-2) caused a global pandemic, resulting in devastating mortality and morbidity [1,2]. Coronaviruses are usually less common pathogens and gained worldwide attention after two major outbreaks: severe acute respiratory syndrome (SARS) in 2002, and Middle East respiratory syndrome (MERS) in 2012 [3]. Following the emergence of the COVID-19 pandemic, more than 500 million confirmed cases and 6 million deaths have been reported worldwide [4]. Several studies have reported the persistence of disabling multisystem symptoms in COVID-19 survivors beyond the initial infection period. This condition is known by multiple terms, such as “long COVID,” “long-haul,” “post-acute COVID syndrome (PACS),” and “post-acute sequelae of SARS-CoV-2 (PASC)” [5,6]. Evolving evidence from the “Household Pulse Survey,” conducted by the U.S. Census and Centers for Disease Control and Prevention (CDC), reported the incidence of long COVID symptoms in one in every five Americans [7]. Groff et al. described the frequency of PASC symptoms at six or more months as 54%, constituting both pulmonary and extrapulmonary manifestations [8]. 

Like other viral infections, SARS-CoV-2 affects both the central and peripheral nervous systems, leading to short- and long-term neurological manifestations [3]. Among the long COVID symptoms listed by the National Institutes of Health (NIH), neurological manifestations such as fatigue, brain fog, anxiety, and depression constitute a higher proportion in COVID-19 survivors. The persistence of these neurological symptoms leads to long-term disability, substantially impacting the quality of life and further adding a significant burden to already strained healthcare systems globally [6].

Although earlier studies examined the prevalence of persistent neurological and neuropsychiatric manifestations in COVID-19 survivors, most of these reports were limited to the initial 3-12 weeks of follow-up [9]. With emerging evidence, there is a need to determine whether the long-term manifestations differ from the short- and medium-term presentations and to assess their impact on disability, health care costs, and the long-term clinical care of these individuals. Our review includes studies with longer follow-up periods, extending up to a year, to analyze the natural history of post-COVID-19 viral complications in terms of incidence, distribution, and progression of the most common neurological and neuropsychiatric symptoms in people with long COVID syndrome.

This article was previously presented as a meeting abstract at the 2024 American Academy of Neurology Annual Meeting on April 14, 2024, and posted to the Research Square preprint server on September 27, 2023.

## Review

Methods

Data Sources and Search Strategy

This systematic review was conducted according to the Preferred Reporting Items for Systematic Reviews and Meta-analyses (PRISMA) guidelines [10]. An electronic search of PubMed/Medline, the Cochrane Trial Register, and Google Scholar was conducted from their inception to April 7, 2023, using the search string: (SARS-CoV-2 OR COVID OR COVID-19 OR coronavirus OR severe acute respiratory syndrome coronavirus 2) AND (neurological OR psychological OR neuropsychological) AND (symptoms OR manifestations) AND (long). In addition, the authors manually screened the cited articles of previous meta-analyses, cohort studies, and review articles to identify relevant studies.

Study Selection

All studies were included if they met the following eligibility criteria described as PECOS: (1) P (Population): population infected with COVID-19; (2) E (Exposure): COVID-19 infection; (3) C (Control): none; (4) O (Outcome): prevalence of long-term neurological and neurocognitive impairment (one year or more) in a population infected with COVID-19; (5) S (Studies): randomized controlled trials, cohort studies, and cross-sectional studies.

Data Extraction

Two reviewers independently searched electronic databases. Studies were exported to the EndNote Reference Library software version 20.0.1 (Clarivate Analytics, Philadelphia, PA, USA), and duplicates were screened and removed. Two investigators independently extracted data and entered it into a computer spreadsheet. Discrepancies were resolved through consensus discussions among investigators. The following data were extracted from each eligible study: first author’s name, population of interest, location of study, study design, sample size, and outcomes.

Quality Assessment of Studies

Two investigators independently assessed the quality of each included study. The Newcastle-Ottawa Scale (NOS) was used to assess the quality of the cross-sectional and cohort studies. A NOS score of 1-5 was considered a high risk for bias, 6-7 was moderate, and a score >7 was considered a low risk of bias. 

Statistical Analysis

The results were formed using a qualitative analysis. We intended to summarize the findings of the articles to synthesize the results. This approach was selected as it incorporated the results from all articles and highlighted the differences and similarities between the study findings. Since the included studies showed heterogeneity among the evaluation criteria and study results, with not all studies containing homogeneous study parameters to carry out a meta-analysis, we decided to conduct a systematic review only.

Results

Literature Search Results

**Figure 1:**
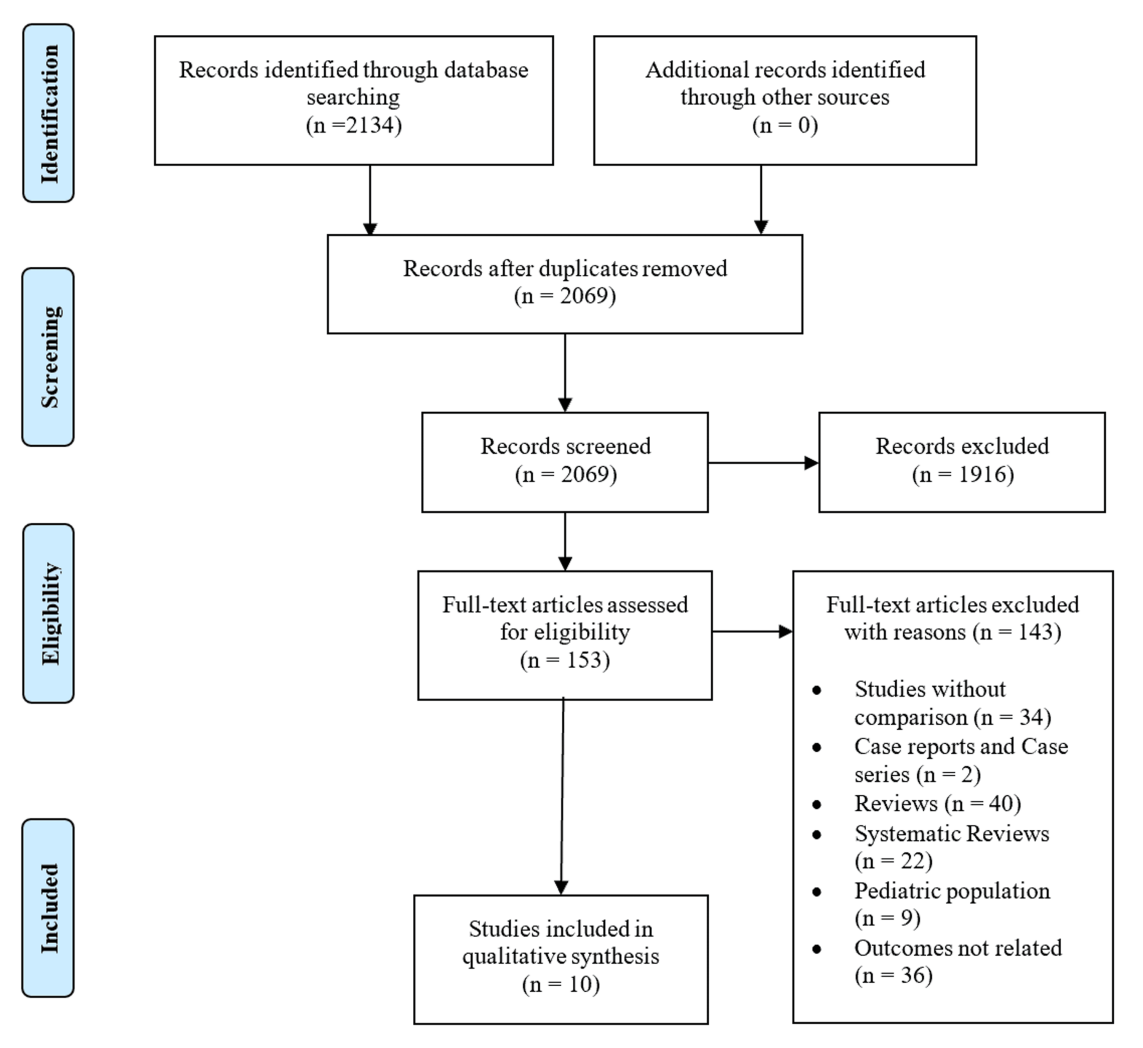
PRISMA flow diagram PRISMA, Preferred Reporting Items for Systematic Review and Meta-analyses

The initial search of the three electronic databases yielded 2,134 potential studies. After exclusions based on titles and abstracts, the full text of 153 studies was reviewed for possible inclusion. After careful screening, 10 studies were included for qualitative analysis [11-20]. Figure 1 summarizes the results of our literature search.

Study Characteristics

Nine prospective cohorts and one cross-sectional study were included in the systematic review (n = 82,987 participants). All the included studies were published from 2021 to 2022 and had an adult population (>18 years). Four studies reported data from China, and six studies were conducted in Europe. The included studies determine the long-term COVID-19 neurological and neurocognitive symptoms. Tables 1-2 provide the baseline characteristics and outcome summaries of the included studies, respectively.

**Table 1:** Baseline characteristics of included studies SARS-CoV-2, Severe acute respiratory syndrome coronavirus-2; HCWs, Healthcare workers; NPS, Nasopharyngeal swab; SARS, Severe acute respiratory syndrome; PTSD, Post-traumatic stress disorder

Author	Study Group	Study Design	Country	Sample Size	Neuro Symptoms Investigated	Outcome	Quality Assessment Rating
Caspersen et al. (2022) [11]	Adults between 35 and 65 years	Cohort study	Norway	73,727	Fatigue, Brain fog, Poor memory, Dizziness, Headache Anxiety, Depression, Mood swings, Altered smell, Altered taste, Sleep problems	At 11-12 months follow up, 16.9% of SARS-CoV-2 infected individuals reported altered smell or taste sensation, with 16.6% of excess risk compared to uninfected individuals; 3.5% of enrolled participants described anxiety, and 4% of individuals reported depression after 1 year; 12% had headache and 17.4% reported fatigue at twelve months following SARS-CoV-2 infection; 9.3% of enrolled subjects experienced sleep problems, and 18.2% of cases reported poor memory. Prevalence of dizziness among enrolled participants was found to be 6% at 1-year follow-up.	Low risk of bias score = 8
Eberst et al. (2022) [12]	Adult SARS-CoV-2 ICU survivors with a median age of 68.4 years	Single-center prospective cohort study conducted at the French University Hospital of Besançon	France	85	Anxiety, Depression	On evaluation at twelve months using the Hospital Anxiety and Depression Scale (HADS), 3.5% of patients were found to have depression symptoms, and 9.6% had symptoms of anxiety.	Moderate risk of bias score = 7
Fortunato et al. (2022) [13]	Adults with mild COVID-19 infection and home quarantined. Mean age of 42.9 ± 17.9 years. Median age is 45 years.	Cross-sectional study	Italy	201	Olfactory dysfunction, Gustatory dysfunction	At twelve months of follow-up, 25.8% of cases (95% CI 19.6-32.9%) described persistent loss of smell, 21.3% (95% CI 15.6-28.1%) of cases had persistent loss of taste; 17.4% (95% CI 12.2-23.8%) reported both smell and taste impairments. The rate of full recovery for olfactory dysfunction improved from 59% at 30 days to 71.9% at 90 days. The recovery rate for gustatory dysfunction increased from 61.3% at 30 days to 74.7% at 90 days.	Moderate risk of bias score = 7
Huang et al. (2021) [14]	Adults with a mean age of 59.0 years (49.0-67.0)	Ambi-directional cohort study	China	1276	Fatigue or muscle weakness, Sleep difficulties, Smell disorder, Taste disorder, Dizziness, Headache, Anxiety, Depression	More patients had anxiety or depression at the 12-month visit vs at the 6-month visit. Women had an odds ratio for fatigue or muscle weakness, anxiety or depression, and diffusion impairment. Matched COVID-19 survivors at twelve months had more problems with mobility, pain or discomfort, and anxiety or depression, and had more prevalent symptoms than controls.	Low risk of bias score = 9
Rivera-Izquierdo et al. (2022) [15]	906 adult patients (>18 years) of which 453 patients were hospitalized due to COVID-19 (exposed) and 453 were hospitalized due to other causes (non-exposed)	Prospective, multicenter cohort study	Spain	906	Headache, Sensitivity disorders, Movement disorders, Confusion, Memory loss	19% of patients were found to have neurologic sequelae at twelve months; 15.5% of patients were found to have mental health sequelae or persistent symptoms.	Low risk of bias score = 8
Strahm et al. (2022) [16]	Healthcare workers with a median age of 40.5 years	Prospective cohort study	Switzerland	3334	Impaired taste/olfaction, Dizziness, Neurocognitive symptoms, Depression, Anxiety	Impaired taste/olfaction and hair loss were the only symptoms that were significantly more common in seropositive HCWs compared with negative controls. SARS-CoV-2 seropositive HCWs without a positive NPS are only mildly affected by long COVID. Neurocognitive symptoms and fatigue symptoms persist, even for those with a diagnosis more than six months ago.	Low risk of bias score = 8
Zhang et al. (2021) [17]	All adult patients with lab confirmed COVID-19 between February 12th and April 10th, 2020	Prospective cohort study	China	2433	Anxiety, Taste change, Impaired sense of smell, Headache	Found that 1095 patients (45.0%) reported at least 1 symptom, and the most common symptoms were fatigue, sweating, chest tightness, anxiety, and myalgia. Compared with male individuals, female patients had a significantly higher percentage of anxiety, myalgia, and headache. Higher levels of stress, depression, and anxiety were also found in female SARS survivors.	Moderate risk of bias score = 6
Zhao et al. (2021) [18]	Adult patients with laboratory-confirmed SARS-CoV-2 infection, and subsequently admitted to the designated local hospitals in Henan Province, were enrolled.	Prospective cohort study	China	94	Insomnia, Headache, Smell disorder, Taste disorder, Dizziness	One year after hospitalization for COVID-19, a cohort of survivors were mainly troubled with muscle fatigue and insomnia. At 1-year follow-up, 61.70% of patients (58 of 94) reported at least one symptom that did not exist before COVID-19 infection. Majority reported muscle fatigue, insomnia, joint pain, headache, hair loss, and chest pain. Eleven patients still experienced a smell or taste disorder.	Moderate risk of bias score = 7
Zhou et al. (2021) [19]	Patients infected with SARS-CoV-2 discharged from Wuhan Union hospital west district (designated hospital for COVID-19) and Fangcang shelter hospitals between January 29, 2020, and April 1, 2020.	Prospective cohort study	China	120	Sleep difficulties, Anxiety, Depression	At the nearly 1-year follow-up, COVID-19 survivors still had multi-system issues, including those in respiratory functioning, radiography, quality of life, and anxiety and depression. Sleep difficulties and fatigue were common symptoms observed during follow-up in one-third of the non-severe cases. A total of 50 (41.7%) and 45 (37.5%) patients reported anxiety and depression, respectively.	Moderate risk of bias score = 6
Rass et al. (2022) [20]	(1) Confirmed SARS-CoV-2 infection; (2) hospitalization or outpatient management with symptoms persisting for at least 6 weeks after initial COVID-19 diagnosis; (3) age greater ≥18 years.	Prospective cohort study	Austria	811	Myalgia, Limb weakness, Headaches, Impaired sensations, Hyposmia, Cognitive deficits, Mental health issues	New and persistent neurological disorders were found. Fatigue, concentration difficulties, forgetfulness, sleep disturbances, myalgia, limb weakness, headache, impaired sensation, and hyposmia were found at 1-year follow-up. Neurological symptoms were found without improvement over time including objective hyposmia. Cognitive deficits were apparent in 18% of participants. Signs of depression, anxiety, and PTSD were found in 6%, 29%, and 10% respectively 1 year after infection.	Moderate risk of bias score = 7

**Table 2:** Summary of outcomes of included studies

	Olfactory Impairment	Taste Impairment	Brain Fog	Anxiety	Depression	Headache	Dizziness	Sleep Disturbance	Fatigue	Impaired Memory
Caspersen et al. (2022) [11]	Trend not reported	Trend not reported	-	Trend not reported	Trend not reported	Trend not reported	Trend not reported	Trend not reported	Trend not reported	Trend not reported
Eberst et al. (2022) [12]	-	-	-	Stable	Stable	-	-	-	-	-
Fortunato et al. (2022) [13]	Improved	Improved	-	-	-	-	-	-	-	-
Huang et al. (2021) [14]	Improved	Improved	-	Worsened	Worsened	Worsened	Stable	Improved	Improved	-
Rivera-Izquierdo et al. (2022) [15]	-	-	Worsened	Stable	Stable	Worsened	-	-	-	Worsened
Strahm et al. (2022) [16]	Improved	Improved	-	-	-	Worsened	Stable	-	Improved	-
Zhang et al. (2021) [17]	Stable	Stable	-	Stable	Stable	Stable	Stable	-	Improved	-
Zhao et al. (2021) [18]	Stable	Stable	-	Improved	Improved	Stable	Stable	-	Improved	-
Zhou et al. (2021) [19]	-	-	-	Stable	Stable	-	-	Stable	Stable	-
Rass et al. (2022) [20]	Improved	-	-	Stable	Stable	Stable	-	Stable	Stable	Stable
Total	7	6	2	7	6	6	4	5	4	4

Results of Quality Assessment

Tables 3-4 provide the quality assessment for the included studies. All studies have a low or moderate risk of bias.

**Table 3:** Quality assessment of included cohort studies

Studies	Selection (Maximum 4)				Comparability (Maximum 2)	Outcome (Maximum 3)			Total Score
	Representativeness of the Exposed Cohort	Selection of the Non-exposed Cohort	Ascertainment of Exposure	Demonstration That Outcome of Interest Was Not Present at the Start of Study	Comparability of Cohorts on the Basis of the Design or Analysis	Assessment of Outcome	Was Follow-Up Long Enough for Outcomes to Occur	Adequacy of Follow-Up of Cohorts	
Caspersen et al. (2022) [11]	1	1	1	1	2	0	1	1	8
Eberst et al. (2022) [12]	1	1	1	1	0	1	1	1	7
Huang et al. (2021) [14]	1	1	1	1	2	1	1	1	9
Rivera-Izquierdo et al. (2022) [15]	1	1	1	1	2	0	1	1	8
Strahm et al. (2022) [16]	1	1	1	1	2	0	1	1	8
Zhang et al. (2021) [17]	1	1	1	1	0	0	1	1	6
Zhao et al. (2021) [18]	1	1	1	1	0	1	1	1	7
Rass et al. (2022) [20]	1	1	1	1	0	1	1	1	7
Zhou et al. (2021) [19]	1	1	1	1	0	0	1	1	6

**Table 4:** Quality assessment of included cross-sectional studies

Studies		Representativeness of the Sample	Sample Size	Non-respondents	Ascertainment of the Exposure (Risk Factor)	Comparability of Cohorts Based on the Design or Analysis			Assessment of the Outcome	Statistical Test	Total Score
Fortunato et al. (2022) [13]	1		1	0	2		0	2		1	7

Results of Qualitative Analysis

A total of 10 studies reported 12-month neurological and neurocognitive symptoms among patients infected with COVID-19 [11-20]. Olfactory and gustatory dysfunction were more prevalent than other symptoms, and these were predominantly reported in individuals with a history of symptoms during acute infection. Anxiety was found to be more prevalent than depression. Fatigue was the most dominant neurocognitive complaint, followed by headache, dizziness, insomnia, and impaired memory.

Olfactory and taste impairment: Seven studies presented data on the incidence of olfactory dysfunction in infected individuals after 12 months. Four studies presented data on impaired taste sensation. Caspersen et al. reported that 16.9% of individuals had altered smell and taste sensations, with a risk ratio of 49.4 (95% CI: 34.5, 70.8) [11]. Fortunato et al. observed a loss of smell in 25.8% (95% CI: 19.6, 32.9) and taste in 21.3% (95% CI: 15.6, 28.1) of individuals. This study showed that sensory symptoms persisted in 3 out of 10 patients after 12 months [13]. Huang et al. found significant improvement in olfactory dysfunction at 12 months (p < 0.0001); however, they reported the symptoms persisted in 4% of individuals. A significant improvement in taste impairment, from 6% at six months to 3% at 12 months, was reported (p < 0.0001) [14]. Strahm et al. found a higher incidence of impaired taste and smell (16% versus 6%) among healthcare workers compared to controls [16]. Zhang et al. reported a 2.5% and 2.2% incidence of severe impairment in the sense of smell and taste, respectively. Additionally, 0.9% of individuals were found to have mild olfactory symptoms, with 1.1% having mild symptoms of taste impairment [17]. Similarly, Zhao et al. reported mild smell and taste disorders in 11.76% of infected patients and severe smell and taste dysfunction in 11.63% of patients. The study showed a statistically non-significant difference between the persistence of symptoms in patients with severe and mild symptoms [18]. Rass et al. observed that 51% of patients had hyposmia after one year [20]. 

Anxiety and depression: Six studies presented data on anxiety and depression. Caspersen et al. reported anxiety in 3.5% and depression in 4% of cohort participants after one year [11]. Using the Hospital Anxiety and Depression Scale, Eberst et al. found a 9.6% prevalence of anxiety and a 3.5% prevalence of depression [12]. Huang et al. reported that 26% of individuals were found to have anxiety or depression [14]. Strahm et al. reported a significant incidence of anxiety and depression in infected healthcare workers (p < 0.001). The Generalized Anxiety Disorder (GAD) score was used to determine the prevalence of anxiety, and the Patient Health Questionnaire was employed to assess depression [16]. Zhang et al. reported severe anxiety in 12.1% of patients, compared to a 10.3% prevalence of non-severe anxiety [17]. Zhou et al. reported a median score of 5 (2-11.5) for anxiety on the Hamilton Anxiety Scale, and a median score of 4 (2-9) for depression on the Hamilton Depression Scale [19]. The Hospital Anxiety and Depression Scale was used by Rass et al.; they found that 29% of patients reported anxiety and 6% reported depression after 12 months [20].

Headache: Caspersen et al. reported an incidence of headache in 12% of enrolled participants [11]. Huang et al. showed a 5% prevalence of headache at 12 months [14]. Rivera-Izquierdo et al. reported a 2.9% prevalence of headache [15]. Strahm et al. found a significant incidence of headaches among infected healthcare workers compared to the non-infected group (p = 0.014) [16]. Zhang et al. reported a 2.3% prevalence of headache - 3.2% in the severe group and 2% in the non-severe group [17]. Zhao et al. reported a total incidence of headaches in 14.89% of patients, with the mild group having 17.65% compared to 11.63% in the severe group. The study showed a statistically non-significant difference between the persistence of symptoms in the severe and mild groups (p = 0.414) [18]. Rass et al. reported an incidence of headaches in 16% of patients [20].

Dizziness: Caspersen et al. found an incidence of dizziness in 6% of enrolled participants [11]. A 5% incidence of dizziness was reported by Huang et al. [14]. Strahm et al. found a non-significant incidence of dizziness among infected healthcare workers after 12 months (p = 0.84) [16]. Zhang et al. reported a 3.3% prevalence of dizziness - 3.8% in the severe group and 3.2% in the non-severe group [17]. Zhao et al. reported a total incidence of dizziness among 10.64% of patients, with the mild group having a 7.84% incidence, compared to 13.95% in the severe group. The study showed a statistically non-significant difference between the persistence of symptoms in the severe and mild groups (p = 0.534) [18]. In a study, Rass et al. reported dizziness in 12% of patients [20].

Sleep disturbance: Caspersen et al. reported a prevalence of sleep disturbance in 9.3% of enrolled participants [11]. Huang et al. reported sleep difficulty in 17% of patients [14]. Rivera-Izquierdo et al. found sleep disturbance in 3.8% of patients [15]. Zhao et al. reported a total incidence of insomnia among 22.34% of patients; the mild group had 19.61%, compared to 25.58% in the severe group. The study showed a statistically non-significant difference between the persistence of symptoms in the severe and mild groups (p = 0.488) [18]. Zhou et al. reported a 43.3% incidence of sleep disturbance - 42.3% in the non-severe group and 50% in the severe group. Statistically insignificant results were reported between both groups (p = 0.563) [19]. Rass et al. reported sleep difficulty in 22% of enrolled patients [20].

Fatigue: Caspersen et al. and Huang et al. reported a 17.4% and 20% incidence of fatigue among enrolled participants [11-14]. Rivera-Izquierdo et al. found that 8.2% of patients had fatigue [15]. Strahm et al. found non-significant tiredness among infected healthcare workers compared to the non-infected group (p = 0.119) [16]. Zhang et al. reported a 27.7% prevalence of fatigue - 35.9% in the severe group and 25.8% in the non-severe group [17]. Zhao et al. reported a total incidence of fatigue in 39.36% of patients; the mild group had a 29.41% incidence, compared to 51.16% in the severe group. The study showed a statistically significant incidence in the severe group (p = 0.032) [18]. Zhou et al. reported a 35.8% incidence of fatigue - 32.7% in the non-severe group and 56.3% in the severe group. Statistically insignificant results were reported between both groups (p = 0.067) [19]. Rass et al. reported fatigue in 38% of patients [20].

Impaired memory: Caspersen et al. reported the incidence of memory impairment as 18.2%, while Rivera-Izquierdo et al. noted a prevalence of memory loss at 3.5% [11-15]. Similarly, Rass et al. identified forgetfulness in 25% of patients [20]. However, most studies, including those by Caspersen et al. and Rass et al., relied primarily on self-reported memory complaints rather than standardized cognitive screening tools, which may limit the reliability of these findings.

Discussion

In this systematic review, we comprehensively reviewed and compared the long-term neurological and neurocognitive manifestations of patients infected with COVID-19 at the 12-month follow-up from 10 studies. Previously, studies have reported the neurological symptoms of COVID-19 infection at three- or six-month follow-ups [21,22]. However, changing disease dynamics mandate periodic epidemiologic revisits of the long-term neurological complications of COVID-19.

In our review, at 12 months, olfactory dysfunction varied from 0.9% to 51%, accounting for the variations in the testing method. For example, studies conducted by Caspersen et al., Fortunato et al., Huang et al., Strahm et al., Zhang et al., and Zhao et al. primarily collected information through symptom questionnaires, meaning the reported percentages of hyposmia are mainly based on self-reported data. This might be influenced by factors such as an individual's awareness of their olfactory deficits or the severity of symptoms they perceive. In contrast, Rass et al. assessed olfactory function using the 16-item Sniffin' Sticks identification test (SS-16; Burghart Medizintechnik), which provides a more standardized and objective measure of olfactory ability.

Additionally, gustatory dysfunction was comparably less prevalent, at 1.1%-11.76%. Nguyen et al. reported a 24% persistence rate of olfactory or gustatory symptoms more than seven months after the onset [23]. Although anosmia and dysgeusia are common neurological manifestations of acute COVID-19, the vast majority recover within 15 days of the resolution of the disease [24]. The mechanisms of persistence could be many. Najafloo et al. pointed out that the angiotensin-converting enzyme 2 (ACE2) receptor expression levels and chronic inflammation are a few of the many cellular and molecular mechanisms involved [25]. Similarly, Raveendran et al. suggested that the varying extent of the injury, the varying time for the recovery of each organ system, the rare persistence of the virus in the body, and an immune response causing the generation of autoantibodies are possible mechanisms for persistence [26].

The data from our systematic review suggest that anxiety is more prevalent (3.5%-29%) at 12 months compared to depression (3.5%-26%). Both disorders are more likely to develop, persist, and increase in frequency post-COVID-19 infection [22]. The severity of depressive symptoms did correlate with systemic inflammation during acute infection in a study by Mazza et al. [27]. In addition to inflammatory and vascular mechanisms, neurotransmitter dysregulation has been hypothesized to play a role in long COVID-related neuropsychiatric symptoms. Alterations in serotonergic, dopaminergic, and glutamatergic systems may contribute to fatigue, mood disturbances, and cognitive dysfunction, although further research is needed to substantiate these hypotheses. Mattioli et al. reported that anxiety, stress, and depression scores were significantly higher in COVID-19 patients than in non-COVID-19 cases at a four-month follow-up [28]. However, the persistence of symptoms at one year indicates the long-term impact of COVID-19 on emotional well-being. The data from our review suggest an urgent need for upscaling psychiatric services, given the vast number of people in the post-COVID-19 phase.

An important limitation in interpreting the prevalence of depression and anxiety in long COVID is the inconsistent reporting of whether these were pre-existing or incident symptoms following infection. While some studies, such as Caspersen et al., attempted to quantify the excess risk in infected individuals compared to controls, many did not report baseline psychiatric status. This lack of clarity limits our ability to distinguish true post-COVID-19 psychiatric sequelae from pre-morbid mental health conditions and may contribute to the overestimation of COVID-19-attributable symptoms. 

We found fatigue to be the most reported neurocognitive symptom, ranging from 8.2% to 56.3%. Headache and dizziness were relatively less prevalent (2%-16% and 3.2%-13.95%, respectively). Sleep disturbances were relatively more prevalent (3.8%-50%), while impaired memory was seen in 3.5%-25%. Previous studies have reported fatigue, sleep disturbances, and impaired memory as common symptoms at three- or six-month follow-ups. We observed similar results at long-term follow-ups. Although these symptoms in the long-term follow-up are likely to be overestimated, similar trends have been reported in large retrospective studies [21,22,29]. It is also important to note that, while some studies, such as Rass et al. and Rivera-Izquierdo et al., reported new-onset neurological symptoms, including headache, most studies did not differentiate between pre-existing and newly developed symptoms. This limitation affects the interpretation of the data, particularly for symptoms like headache, which have a known baseline prevalence in the general population.

Silva Andrade et al. explained the possible molecular mechanisms responsible for long COVID symptoms [30]. The neurotrophic and neuroinvasive properties of the virus are due to the high expression of the human angiotensin-converting enzyme-2 receptor (hACE2-R) in the brain, allowing the virus to cross the blood-brain barrier easily [31]. In the long term, systemic innate-mediated hyperinflammation, residual viral particles causing a low-grade inflammatory response, and direct or indirect nervous system damage during the acute phase are the potential pathophysiological mechanisms responsible for the lingering neurological effects [32,33]. Clinicians should also take note of the significant overlap of neuropsychological symptoms of acute respiratory distress syndrome and long COVID, which could overestimate the results [34].

Recent literature has proposed several additional pathophysiological mechanisms contributing to long COVID. These include persistent viral reservoirs that evade immune clearance, chronic immune activation leading to immune dysregulation, and microvascular dysfunction causing ongoing tissue injury. These emerging theories warrant more in-depth investigation to develop targeted therapeutic strategies. From a clinical management standpoint, our findings underscore the importance of early identification and monitoring of patients with persistent neurological symptoms. Screening protocols for cognitive impairment, anxiety, depression, and fatigue should be integrated into post-COVID-19 care pathways. Rehabilitation strategies - ranging from cognitive behavioral therapy to physical and occupational therapy - may be beneficial, particularly when guided by multidisciplinary teams.

As suggested by the data above, the prevalence of each neurological symptom in our study was found to be highly variable. Hence, the heterogeneous nature of the data from the existing studies is the main limitation of our study. Various factors could contribute to this variability, including (1) inconsistent definitions of the symptoms reported across studies, (2) studies involving patients with varying COVID-19 severity and a lack of standardized follow-up protocols, (3) variable sample sizes, (4) confounding due to studies involving patients of different ethnicities from various countries across Europe and China, and (5) age-based bias resulting from studies including patients across diverse adult and older age groups, leading to variable mean ages in each study. Another limitation is the overestimation of the true prevalence of clinically significant long COVID symptoms due to the lack of control groups in several studies. Furthermore, most of the included studies assessed memory impairment through patient self-report rather than using standardized cognitive screening tools. This introduces potential bias and variability in symptom reporting, highlighting the need for objective cognitive assessments in future studies.

Long COVID is a complex spectrum of symptoms, including neurological and neuropsychiatric manifestations. In our study, we aim to fill the gap in knowledge regarding the prevalence of these symptoms at long-term follow-up. However, further research is needed to better understand the underlying mechanisms responsible for the persistence of symptoms after COVID-19. Additionally, there is a need to develop a standardized, multidisciplinary approach to mitigate the symptoms of long COVID during both the acute infection phase and follow-up.

While long COVID is increasingly recognized as a condition that spans multiple organ systems, our review did not identify studies specifically assessing the role of a multidisciplinary approach in reducing the neurological symptom burden. Given the complex interplay between psychiatric, neurological, and general medical symptoms, future research should evaluate whether multidisciplinary models involving neurology, psychiatry, physical medicine and rehabilitation, and primary care can improve outcomes and reduce the persistence of symptoms.

## Conclusions

In our study, at one-year follow-up, we found fatigue to be the most reported neurocognitive complaint, affecting up to 56% of individuals. While variable, olfactory dysfunction and sleep disturbances were observed in up to half of the patients, and anxiety, depression, and memory dysfunction were found in up to a quarter of patients. Future studies should further delineate the mechanisms behind the neurological symptoms of long COVID and provide deeper insights into potential treatment strategies. Additionally, understanding the underlying neurobiological mechanisms of long COVID can aid in the identification of biomarkers for early detection and management of the condition. Longitudinal studies with larger sample sizes and standardized assessments can provide more reliable estimates of the prevalence and nature of neurocognitive symptoms in long COVID, as well as the factors that contribute to their persistence or resolution over time. Moreover, research should focus on identifying risk factors for developing long COVID, such as genetic, environmental, and social determinants, to inform preventive interventions and optimize healthcare delivery for this growing population along with improvement of quality of life for patients with these symptoms.
